# A Novel Machine Learning Model for Dose Prediction in Prostate Volumetric Modulated Arc Therapy Using Output Initialization and Optimization Priorities

**DOI:** 10.3389/frai.2021.624038

**Published:** 2021-04-23

**Authors:** P. James Jensen, Jiahan Zhang, Bridget F. Koontz, Q. Jackie Wu

**Affiliations:** Department of Radiation Oncology, Duke Cancer Institute, Durham, NC, United States

**Keywords:** dose prediction, multi-criterial optimization, treatment planning, prostate VMAT, machine learning, artificial intelligence, residual neural networks

## Abstract

Treatment planning for prostate volumetric modulated arc therapy (VMAT) can take 5–30 min per plan to optimize and calculate, limiting the number of plan options that can be explored before the final plan decision. Inspired by the speed and accuracy of modern machine learning models, such as residual networks, we hypothesized that it was possible to use a machine learning model to bypass the time-intensive dose optimization and dose calculation steps, arriving directly at an estimate of the resulting dose distribution for use in multi-criteria optimization (MCO). In this study, we present a novel machine learning model for predicting the dose distribution for a given patient with a given set of optimization priorities. Our model innovates upon the existing machine learning techniques by utilizing optimization priorities and our understanding of dose map shapes to initialize the dose distribution before dose refinement via a voxel-wise residual network. Each block of the residual network individually updates the initialized dose map before passing to the next block. Our model also utilizes contiguous and atrous patch sampling to effectively increase the receptive fields of each layer in the residual network, decreasing its number of layers, increasing model prediction and training speed, and discouraging overfitting without compromising on the accuracy. For analysis, 100 prostate VMAT cases were used to train and test the model. The model was evaluated by the training and testing errors produced by 50 iterations of 10-fold cross-validation, with 100 cases randomly shuffled into the subsets at each iteration. The error of the model is modest for this data, with average dose map root-mean-square errors (RMSEs) of 2.38 ± 0.47% of prescription dose overall patients and all optimization priority combinations in the patient testing sets. The model was also evaluated at iteratively smaller training set sizes, suggesting that the model requires between 60 and 90 patients for optimal performance. This model may be used for quickly estimating the Pareto set of feasible dose objectives, which may directly accelerate the treatment planning process and indirectly improve final plan quality by allowing more time for plan refinement.

## Introduction

Volumetric modulated arc therapy (VMAT) is a cancer treatment option that can effectively irradiate a target while minimizing the nearby healthy tissue irradiation in relatively short delivery times (Otto, 2008; Teoh et al., 2011). Dual-arc VMAT has been shown to be an effective treatment technique for prostate cancer (Guckenberger et al., 2009; Zhang et al., 2010). VMAT treatment planning relies on inverse planning techniques that perform dose optimization and dose calculation to create a deliverable treatment plan. To employ existing single-function minimization algorithms, VMAT optimization techniques typically scalarize the dose objectives into a weighted sum to use as the optimization loss function, with the weights (priorities) decided by a treatment planner. Dose objective scalarization allows the treatment planner to create and evaluate several plans by providing multiple priority combinations to the optimizer to create a subjectively optimal treatment plan. This problem can be formulated as a multi-criteria optimization (MCO) problem, in which the treatment planner has to learn about the set of feasible plan doses which cannot be strictly improved, which is historically named, the Pareto surface (Hwang and Masud, 1979; Miettinen, 1999). MCO has been studied extensively and many methods for exactly sampling the Pareto surface have been implemented for radiation therapy treatment planning systems (Craft et al., 2007; Monz et al., 2008; Bokrantz and Forsgren, 2013).

However, these contemporary MCO methods ultimately require the generation of many treatment plans to sample the Pareto surface. In this framework, the treatment planner samples the Pareto surface and linearly interpolates the sampled plans to infer the feasible ranges of dose trade-offs. However, VMAT treatment planning using current commercial treatment planning systems can take 5–30 min per plan to optimize and calculate, so that the exact methods for sampling the Pareto surface can take a longer time to run. This time cost reduces the remaining amount of time that the planner has for manual plan refinement and also limits the precision of the surface sampling, decreasing the accuracy of any subsequent surface interpolations and limiting the understanding of the planner with regard to feasible dose trade-offs. All these factors combine to reduce the quality of the final treatment plan.

The primary goal of this study is to present a method for quickly estimating the dose distribution for a given set of optimization priorities. This method would be able to quickly and accurately estimate the Pareto surface for a given patient and indirectly improve the quality of the final plan by allowing the treatment planner more time for plan refinement.

In recent years, machine learning has seen success in image classification and processing tasks, due to the ability of modern convolutional neural network variants, such as residual networks (ResNets) and U-Nets, to quickly detect and manipulate learned image patterns (Simonyan and Zisserman, 2014; Ronneberger et al., 2015; He et al., 2016). Inspired by the speed and accuracy of these results, we hypothesized that it was possible to use a similar model to bypass the time-intensive dose optimization and dose calculation steps in treatment planning, arriving directly at the resulting dose distribution and computing the relevant dose objectives. Such a model would greatly benefit the treatment planning system (TPS), as it would provide a way to quickly estimate the dose distributions of many treatment plans to infer the Pareto surface of a given patient for feasible dose objectives.

In this study, we present a novel machine learning model for predicting the TPS-simulated dose distribution for a given patient. Similar models have previously been implemented which are more directly drawn from the U-Net architecture (Babier et al., 2019; Nguyen et al., 2019), but these models have undergone only modest modification for the specific task of dose prediction. The primary motivation behind our model is to use our understanding of the general shape of dose distributions to remove much of the non-linearity of the dose prediction problem and decreasing the difficulty of subsequent network predictions. Our model takes the optimization priorities of the treatment plan, which were taken into account during dose prediction, and infers feasible dose distributions across a range of optimization priority combinations, allowing for indirect Pareto surface inference. This model is also relatively fast (0.05 s per plan), and it is capable of sampling the entire Pareto surface much faster than commercial dose optimization and dose calculation engines.

## Methods

### Patient Cohort and Treatment Planning Technique

Hundred prostate cancer patients were retrospectively included in this study. The data of each patient consisted of an abdominal CT scan and contours of their planning target volume (PTV), the bladder, the rectum, the left femoral head, and the right femoral head. After anonymization, patient datasets were imported to a commercial treatment planning system for dose optimization and dose calculation. The PTV dose prescription was set to 70 Gy in 29 fractions, as is the current standard for clinical practice at our institution. During treatment planning, each plan included two concentric, coplanar VMAT beams centered on the PTV, with field sizes set to encompass the PTV during a 358-degree beam rotation. Beam collimators were set at 15° and 345° to reduce the effect of collimator leaf gap overlap. During optimization, priorities were placed on the PTV homogeneity index (HI = D2%–D98%), bladder D25%, and rectum D25%. These objectives were chosen to represent the dimensions of trade-off during treatment planning, since the primary goals of prostate VMAT are uniform PTV coverage, bladder sparing, and rectum sparing. These objectives had different optimization priority combinations for each plan to sample the Pareto surface of dose trade-offs. After optimization, plans were normalized such that PTV D95% equaled 100% of the dose prescription of the target. Fixed constraints for each plan optimization included PTV D93% < 101% to reduce the dose-shifting effect of plan normalization, as well as D0.01cc < 65% for both femoral heads in accordance with the standard practice of our institution for normal critical structure constraints. Variable constraints included PTV HI < 10%, the bladder D25% < 30% of prescription, and the rectum D25%. For each patient, the Pareto surface was sampled by optimizing and calculating 25 plans that follow. Each plan had a different optimization priority combination and therefore sampled a different location on the Pareto surface. Bounding points on the surface were chosen through manual plan optimization such that the bounding points represented clinically feasible plans. Subsequent points on the surface were created using linear combinations of the objective priorities of the bounding points; this ensured that all interior points also represented clinically feasible plans on the Pareto surface. Beamlet fluence optimization and dose calculation were performed with the commercial treatment planning system. After each plan was calculated, the corresponding dose map, critical structure maps, and optimization priority combination were exported for use during model training and evaluation.

### Dose Prediction Model Architecture

**Figure 1:**
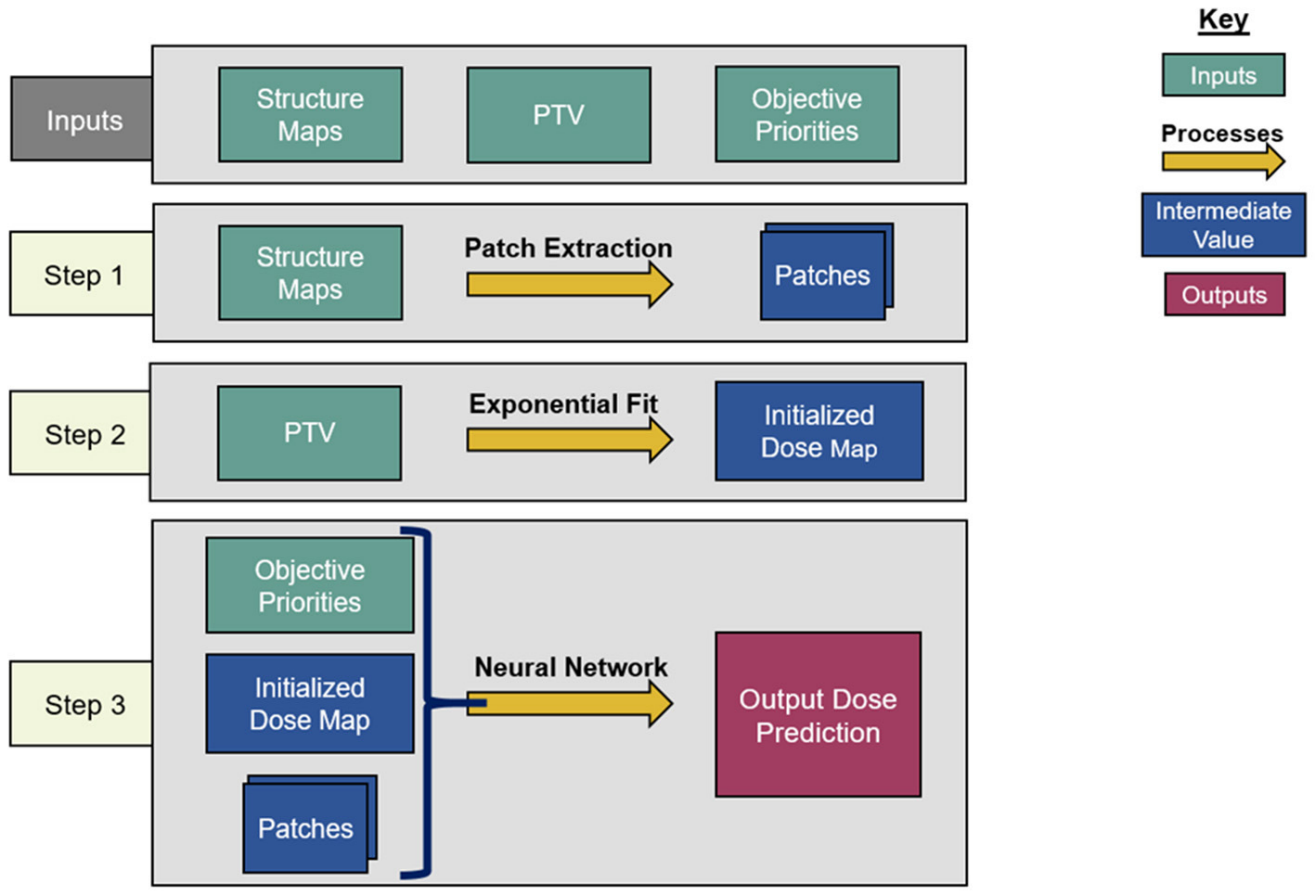
Overview of the dose prediction model architecture.

An overview of the architecture of the dose prediction is depicted in Figure 1. The inputs of the model are the objective priorities and structure maps of the PTV, the bladder, and the rectum, resized to slices of 128 × 128 voxels to increase model efficiency. These structure maps are binary image-domain representations of the corresponding structures, indicating for each pixel whether that pixel is inside the contour of the structure. These structure maps have been scaled by the objective priorities of their corresponding structure for each plan. This is a straightforward way to incorporate objective trade-off priorities without complicating the architecture of the model.

#### Dose Initialization

The model begins by creating an initial dose distribution *via* an inverse fit of inter-slice and intra-slice PTV distance maps on a voxel-wise basis. The functional form of the initialized dose fit is as follows:

$$\begin{array}{l} D_{i}={\left[1+a_{1}\;*\;ISD_{1}^{a_{2}}\;+c\;*\;ISD_{2}^{a_{3}}\right]}^{-1} \end{array}$$

where *ISD*_1_ refers to the inter-slice distance from the voxel to the nearest PTV location within the slice of the voxel, *ISD*_2_ refers to the intra-slice distance from the voxel to the nearest PTV location at the row and column of the voxel, and *a*_1_, *a*_2_, and *a*_3_ are variables that need to be fitted. The purpose of this initialization is to allow the subsequent neural network to predict the shift between the initialization and the TPS-simulated dose distribution rather than the dose distribution itself. We hypothesize that these shifts are more likely linear than the dose distribution itself and therefore more easily learned.

#### Patch Extraction

The model proceeds by extracting three sets of 9 × 9 transverse patches from all structure maps and the initialized dose map at each voxel. Each set of patches has a different atrous rate, which is the number of voxels skipped between the sampled voxels. The first patches have an atrous rate of 1, i.e., they do not skip any voxel and are contiguous. These patches allow the model to infer local structure information near the pixels on which they are centered. For the second patches, the structure maps are smoothed by convolution with a uniform 3 × 3 kernel, and the patches are extracted with an atrous rate of 3. Similarly, the third patches are extracted with an atrous rate of 10 from the structure maps after smoothing by a 10 × 10 kernel. The smoothing convolutions are performed to make each voxel within the atrous patches contain structure information from the nearby voxels that the atrous sampling skips.

The idea of atrous convolution (also called, dilated convolution) was originally presented by Yu and Koltun (2015). The motivation for including multiple patches with different atrous rates is to capture the features of the input data at both coarse and fine levels. This removes the need for traditional downsampling and upsampling layers in the network. For the patches with atrous rates >1, the combination of average smoothing and atrous sampling essentially increases the receptive field size per layer of the model, so that the model can infer the effect of critical structures at both large and short distances without significantly increasing the amount of memory or model parameters required. It is to be noted that the model architect can choose the number of patches and the atrous rates of every patch, and a similar model with atrous rates near 1, 3, and 10 will produce results similar to the result of this model. For this model, the atrous rates were chosen based on the nature of the input data. The patches with an atrous rate of 1 captured every fine detail, the patches with an atrous rate of 10 spanned most of the images and captured every coarser detail, and the patches with an atrous rate of 3 reflected the more intermediate features. The patches are then cast into 81-element vectors per voxel, and the vectors and optimization priorities are all concatenated voxel-wise to serve as input for the residual network.

#### Residual Network

The model then uses the patch vectors as inputs for a neural network, which is inspired by the recently developed ResNet (He et al., 2016). This network is used to determine an update to the dose initialization rather than computing the dose from scratch. The natural choice for the construction of this intermediate network was the residual network (ResNet), because the residual blocks of ResNets were originally designed with a similar concept in mind. As explained by He et al., residual blocks tend to perform much better than the conventional network blocks at a higher depth when the effects of the input features resemble linear residuals. This happens partially because the residual formalism makes the gradients to be less susceptible to vanishing or exploding, improving the convergence (He et al., 2016). Unpublished internal testing confirmed that the performance of our model degrades when it replaces the residual blocks with standard convolutional or fully connected (FC) blocks. Moreover, residual blocks have been shown to make the performance of the model less dependent on the number of blocks included, which reduces the need for fine-tuning the number of blocks in the model.

**Figure 2:**
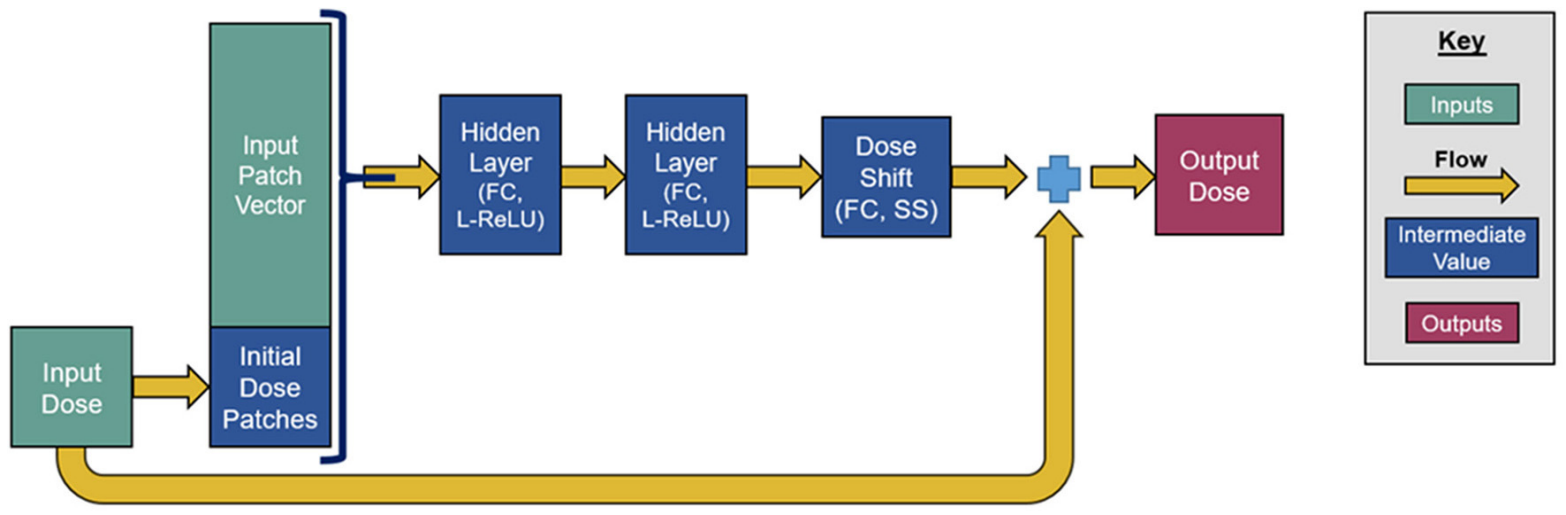
Graphical depiction of a residual block within the neural network.

Our network consists of a series of six residual blocks that sequentially update the initialized dose map. Each residual block, depicted in Figure 2, consists of three FC layers. The first two layers have 100 output units and leaky rectified linear unit (L-ReLU) activations are defined as follows;

$$\begin{array}{l} L-ReLU\;\left(x\;\right)=x\text{ when }x\;>\;0\text{ and }L-ReLU\;\left(x\;\right)\\ \;=0.2x\text{ when }x\leq 0. \end{array}$$

These first two layers extract quasi-linear features from the patch vectors. The last layer has a single output and scaled softsign (SS) activation, defined as, *SS*(*x*) = 0.3*x*/(1+|*x*|). The purpose of this last activation function is to take the quasi-linear combinations from the previous layer and map them to a suitable dose shift with a limited range. Since each residual block changes the initialized dose map, the dose map patches need to be reextracted after each update. The number of residual blocks, layers per block, and output units per layer were chosen somewhat subjectively, and we anticipated that the accuracy achieved by this neural network can be achieved through similar network designs and hyperparameter tunings.

### Model Training

The training loss function was the root-mean-square error (RMSE) between the predicted dose map and TPS-simulated dose map, restricted to voxels within the body contour and restricted to slices containing at least one critical structure. Dose initialization variables were fit according to the RMSE between the initialized dose map and the TPS-simulated dose, and these variables were trained before the residual network variables. Gradients for the loss function were estimated using batches of training data, with each batch containing several slices approximately equal to the typical number of slices that a patient would have. Slices in the batches were sampled diagonally, such that the batch slices were located at different levels within different patients. This sampling means that each batch contains slices from most patients in the training set at most slice positions, such that each batch is a good representation of the entire cohort. Therefore, the gradients computed from the batches were in close approximations to the gradients of the loss function applied to the entire cohort, improving the optimization of convergence and stability. The model was trained using the Adam optimization algorithm, which was designed for stochastic gradient-based optimization (Kingma and Ba, 2014). Kingma and Ba recommend specific hyperparameters, including step size α = 0.001, decay hyperparameters β_1_ = 0.9, and β_2_ = 0.999, and error epsilon ϵ = 10^−8^, all of which are used in the training of our model. The Adam optimizer is particularly appropriate here because the batch gradient computations are stochastic. The trainable parameters in each layer were initialized using the Glorot uniform initializer, which initializes variables by sampling randomly from a uniform distribution bounded by $\pm \sqrt{6\;/\left(number\;of\;inputs+number\;of\;outputs\right)}$ (Glorot and Bengio, 2010). The Glorot uniform initializer was designed to model the inherent variance of rectified linear unit activation functions, similar to the activation functions used in our residual network. All aspects of the model, including optimization and evaluation, were implemented using the Tensorflow machine learning platform with an NVIDIA Quadro M4000. Optimization proceeded for 2,000 iterations before termination.

### Predicted Pareto Surface Evaluation

Pareto surfaces are generated from the model by passing several optimization priority combinations as inputs and evaluating the relevant dose-volume metrics from the resulting dose maps. For analysis, this study compared the Pareto surfaces of the clinical and predicted dose maps using the same optimization priority combinations for both surfaces, allowing for direct comparison of matched plans which should produce the same dose maps and objective metrics. However, when evaluating the accuracy of a predicted Pareto surface, we were more interested in the entire surface as a connected set rather than a few points which sample the surface. Although we can use the sampled points to interpolate the Pareto surface, distances between the sampled points do not necessarily represent the distances between points which are interpolated from the sampled points (Jensen et al., 2020). For this reason, we believe that it is insufficient to simply evaluate the RMSEs between the sampled points in Pareto space as a metric for the closeness of the represented predicted Pareto surface to the TPS-simulated Pareto surface. To our knowledge, no previous publications on dose prediction for radiation therapy have directly evaluated the distance between the Pareto surfaces generated by their models.

To overcome this insufficiency, we tested three metrics in addition to RMSE between the matched points in Pareto space. The first additional metric is the Hausdorff distance, mathematically defined between the two sets *A* and *B* are as follows:

$$d_{H}(A,B)=max\;\left\{\underset{x\in A}{sup}\;\underset{y\in B}{inf}|x-y|,\underset{y\in B}{sup}\;\underset{x\in A}{inf}|x-y|\right\}$$

where *A* and *B, in this case*, represent the vertices (sampled points) of each Pareto surface. One benefit of the Hausdorff distance is that it is sensitive to outliers so that the Hausdorff distance between the sets of Pareto surface vertices should be similar to the Hausdorff distance between the actual Pareto surfaces as interpolated sets. However, this sensitivity to outliers causes Hausdorff distances to represent the maximum error rather than the average error more strongly. In the context of machine learning, this is a drawback because the outliers which influence the Hausdorff distance can fluctuate because of the random initial conditions of the model.

**Figure 3:**
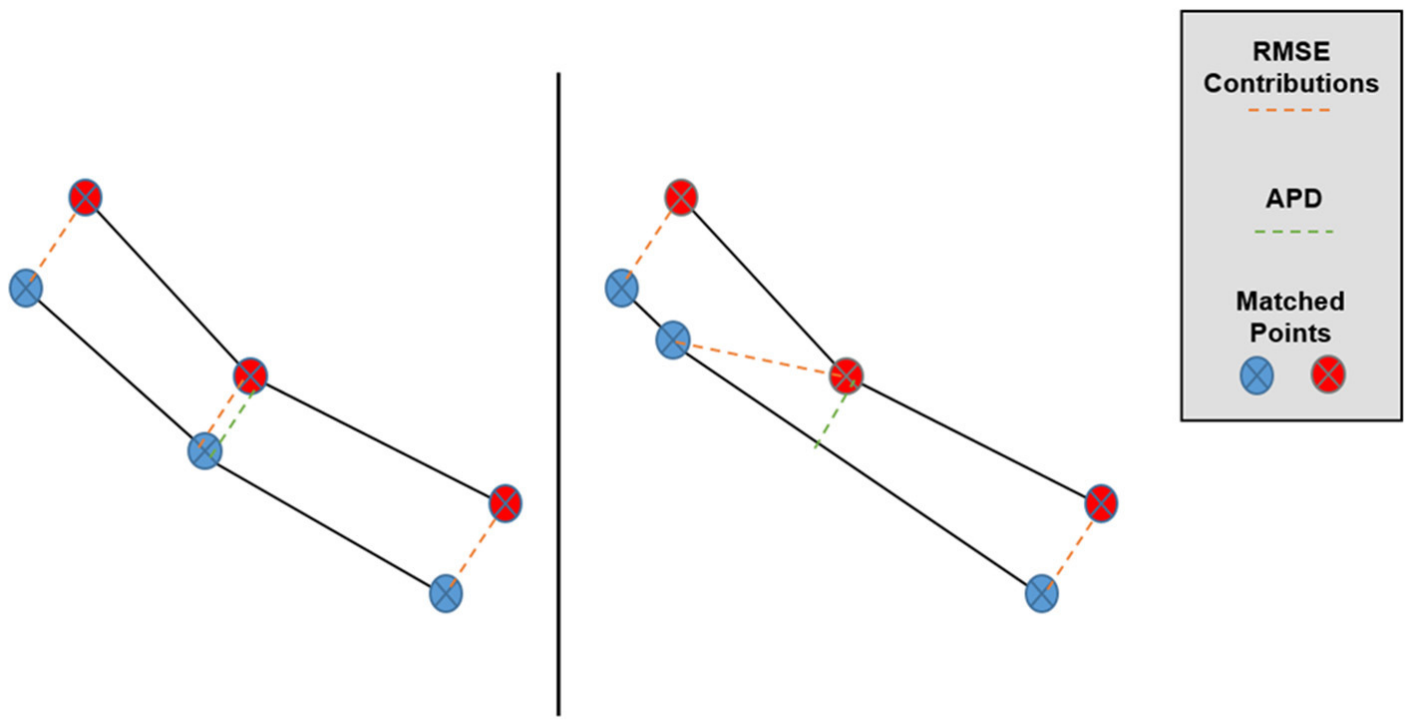
Graphical depiction of the effect of matched point error along the Pareto surfaces on RMSE and APD. Despite the lower surfaces being very similar, the central matched pair has a much larger contribution to Pareto RMSE in the right case while the APD remains approximately the same.

The second additional metric is the average projected distance (APD) in Pareto space, which addresses some of the insufficiencies of Pareto space RMSE and Hausdorff distance. A more abstract discussion has been published about the properties of the APD and why this metric is superior to the RMSE in Pareto space (Jensen et al., 2020). The APD examines the vector displacements between matched points between two sets and then averages the displacements when projected along the direction of the average vector displacement. The APD between two sets, *A* and *B* is mathematically defined as follows:

$$\begin{array}{l} APD\left(A,B\right)=E\left[\left(x_{i}-y_{i}\right)\cdot E\left[x_{i}-y_{i}\right]\right]/\left|E\left[x_{i}-y_{i}\right]\right| \end{array}$$

where the (*x*_*i*_, *y*_*i*_) symbols enumerate the matched pairs of points between sets, A and B (in our case, the TPS-simulated and predicted Pareto surfaces), and E refers to taking an average over these matched pairs for one patient. The primary motivation behind the APD as a Pareto space metric is depicted in Figure 3, in which we can see that overall Pareto surface interpolation accuracy is not affected by the pointwise error components along the respective Pareto surfaces. APDs first remove these error components, so we expect the APD to better measure the closeness of the interpolated Pareto surfaces compared to the RMSE or HD.

The third metric is the average nearest point distance (ANPD) in the Pareto space, which supersamples the simplicial complex representations of the Pareto surfaces and averages the distance from each sampled point of one surface to the simplicial complex of the other surface. The ANPD between the two sets, *A* and *B* is mathematically defined as follows:

$$ANPD\left(A,B\right)=avg\;\left\{\underset{\text{y}\epsilon \text{S}(\text{B})}{avg}\;\underset{\text{x}\epsilon \text{S}(\text{A})}{inf}{\left\|x-y\right\|}_{2},\;\underset{\text{x}\epsilon \text{S}(\text{A})}{avg}\;\underset{\text{y}\epsilon \text{S}(\text{B})}{inf}{\left\|x-y\right\|}_{2}\right\}$$

where the sets *A* and *B* represent the vertices of each Pareto surface and *S*(*A*) and *S*(*B*) represent the simplicial complexes spanned by the vertices of *A* and *B*, respectively. Here, each Pareto surface vertex corresponds to one dose distribution generated by different optimization priorities. The primary motivation behind the ANPD as a Pareto space metric is that it reflects the individual distances from each point on one surface to the other surface, which we imagine to be the “true” distance between that point and the surface. Like the APD, the ANPD has already been discussed according to its properties and comparison to the RMSE in another publication (Jensen et al., 2020). For this publication, all these metrics are presented because a consensus about the optimal metric has not yet been established.

### Model Evaluation

When evaluating this model, a single instance of training and testing the model is insufficient because the performance of the model depends on the specific training set and testing set. To counteract the randomness associated with choosing a training set and testing set, the following evaluation scheme was used. After the model was designed and developed, it was evaluated using a 10-fold cross-validation repeated 50 times. In this evaluation, one repetition of 10-fold cross-validation involves randomly partitioning the patient dataset into ten 10-patient subsets and training the model 10 times, with each training set using a different subset for testing and the rest of the subsets for evaluation. In one repetition of 10-fold cross-validation, each patient appears in training sets exactly nine times, and each patient appears in the testing sets exactly once. Therefore, the 10-fold cross-validation partially negates the effect of randomly assigning patients into training and testing sets. In this evaluation, 10-fold cross-validation was repeated 50 times, with the patient dataset partitioned into different subsets for each repetition. By repeating the cross-validation many times, the randomness associated with the random selection of the training and testing sets is reduced further. Overall, this evaluation involved training and testing the model 500 times, which is 10 training/testing pairs for each of the 50 cross-validation repetitions. The results from all 500 model validations were aggregated by the training set and the testing set using the metrics described in the sections above.

To test the performance of the model with smaller training datasets, another set of cross-validations was performed using different ratios of training data to testing data. These cross-validations evaluated the performance of the model with training-to-testing data set ratios of 90%:10%, 80%:20%, 70%:30%, 60%:40%, 50%:50%, 40%:60%, 30%:70%, 20%:80%, and 10%:90%. For example, the second of these cross-validations used 80 patients to train each model and 20 patients to test each model. For this cross-validation, the patients were grouped into ten 10-patient subsets, enumerated #1, #2, #3, #4, #5, #6, #7, #8, #9, and #10. The first model validation in the cross-validation was trained on subsets #1-8 and tested on subsets #9 and #10; the second validation was trained on subsets #2-9 and tested on subsets #10 and #1; the third validation was trained on subsets #3-10 and tested on subsets #1 and #2; and so on. In this way, each of the cross-validations at smaller training-to-testing ratios evaluated the model ten times. This is not strictly a 10-fold cross-validation, but it is a cross-validation because every patient appears in the same number of training subsets and the same number of testing subsets. For comparison, the cross-validation with an 80%:20% ratio evaluates 10 trainings of the model, while normal 5-fold cross-validation evaluates 5 trainings of the model. The utility of this approach can be seen clearly for the 50%:50% case, where the corresponding 2-fold cross-validation only involves training the model twice. Clearly, this is not thorough enough, but the other extremity of testing every possible 50%:50% partition of the data is not feasible due to time constraints. This approach of cycling through the ten subsets strikes a compromise between thoroughness and efficiency when evaluating the model at smaller training set sizes. For each cross-validation, the performance of the model on the training and testing sets was aggregated to evaluate the performance of the model while reducing the randomness associated with grouping the patients into training and testing subsets.

## Results

### Direct Dose Map Evaluation

**Figure 4:**
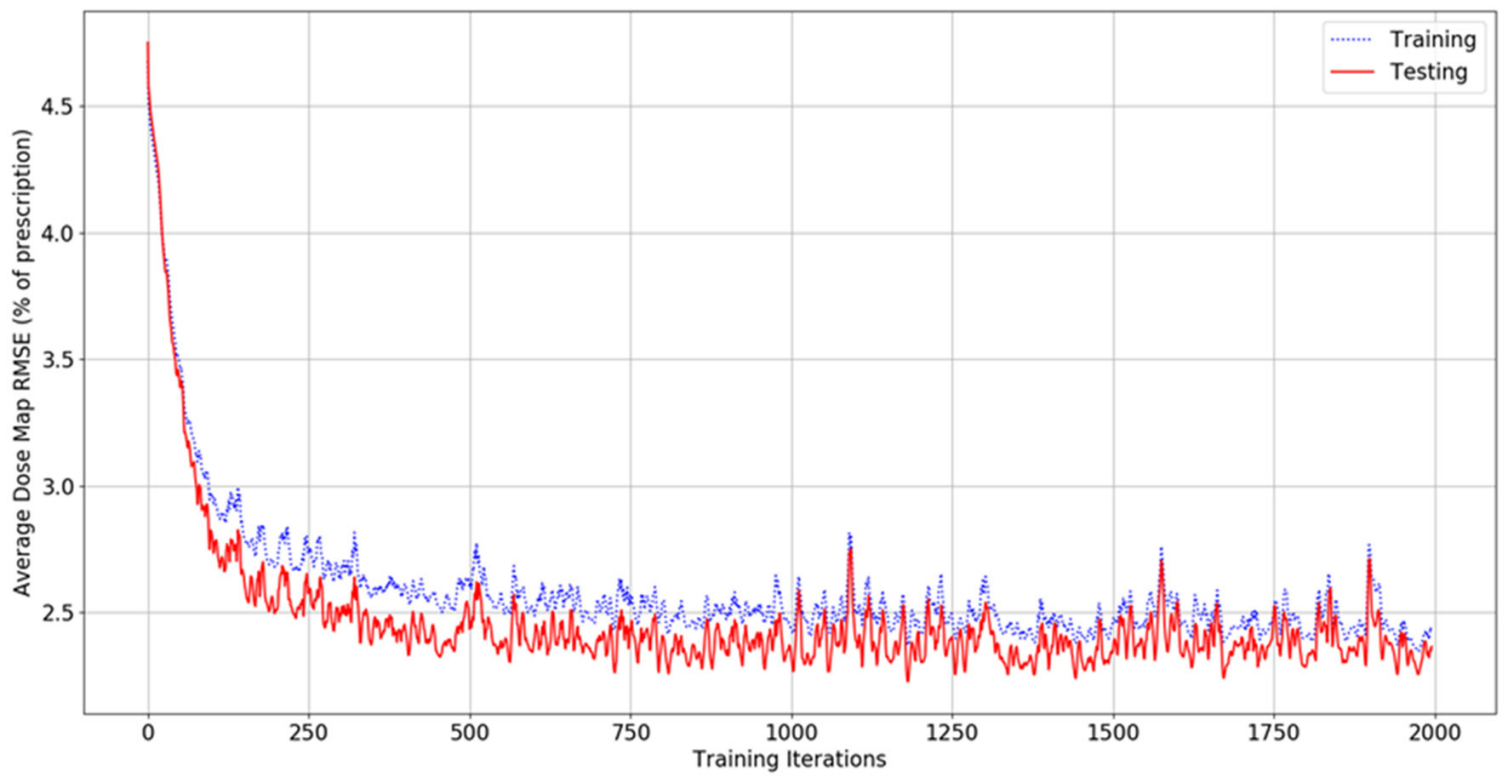
Graph of dose map root-mean-square error for the training set (blue, dashed) and testing set (red, solid) as a function of the number of iterations during model training.

Figure 4 shows the dose map RMSE for the aggregated training and testing sets during a randomly selected model instance training. After training, the mean dose map RMSEs were 2.44 ± 0.89% and 2.42 ± 0.47% for the training and testing set dose predictions, respectively, across all cross-validations. These errors demonstrate that the model can achieve good prediction accuracy on a voxel-by-voxel basis. The difference between the training and testing dose map RMSEs is less than their standard deviations, suggesting that the performance of the model is similar for both the training and testing datasets. The dose map RMSEs due to the initialization fit alone were 5.12 ± 0.55%, and 5.54 ± 1.30% for the training and testing sets, respectively. This indicates that the residual network makes a measurable improvement to the dose initialization and that the model successfully learns after the dose initialization. Note that these values differ from the general dose map RMSE of the model at 0 iterations into training because the residual parameters and effects of the network on prediction are non-zero and initialized randomly. For comparison, the International Commission on Radiation Units and Measurements (ICRU) and Task Group 142 of the American Association of Physicists in Medicine (AAPM) have stated that a 5% maximum dosimetric uncertainty is appropriate for standard intensity-modulated radiation therapy (IMRT) treatments (ICRU, 1976; Klein et al., 2009). Therefore, these dose map RMSEs are comparable to the maximum error permitted in treatment delivery.

**Figure 5:**
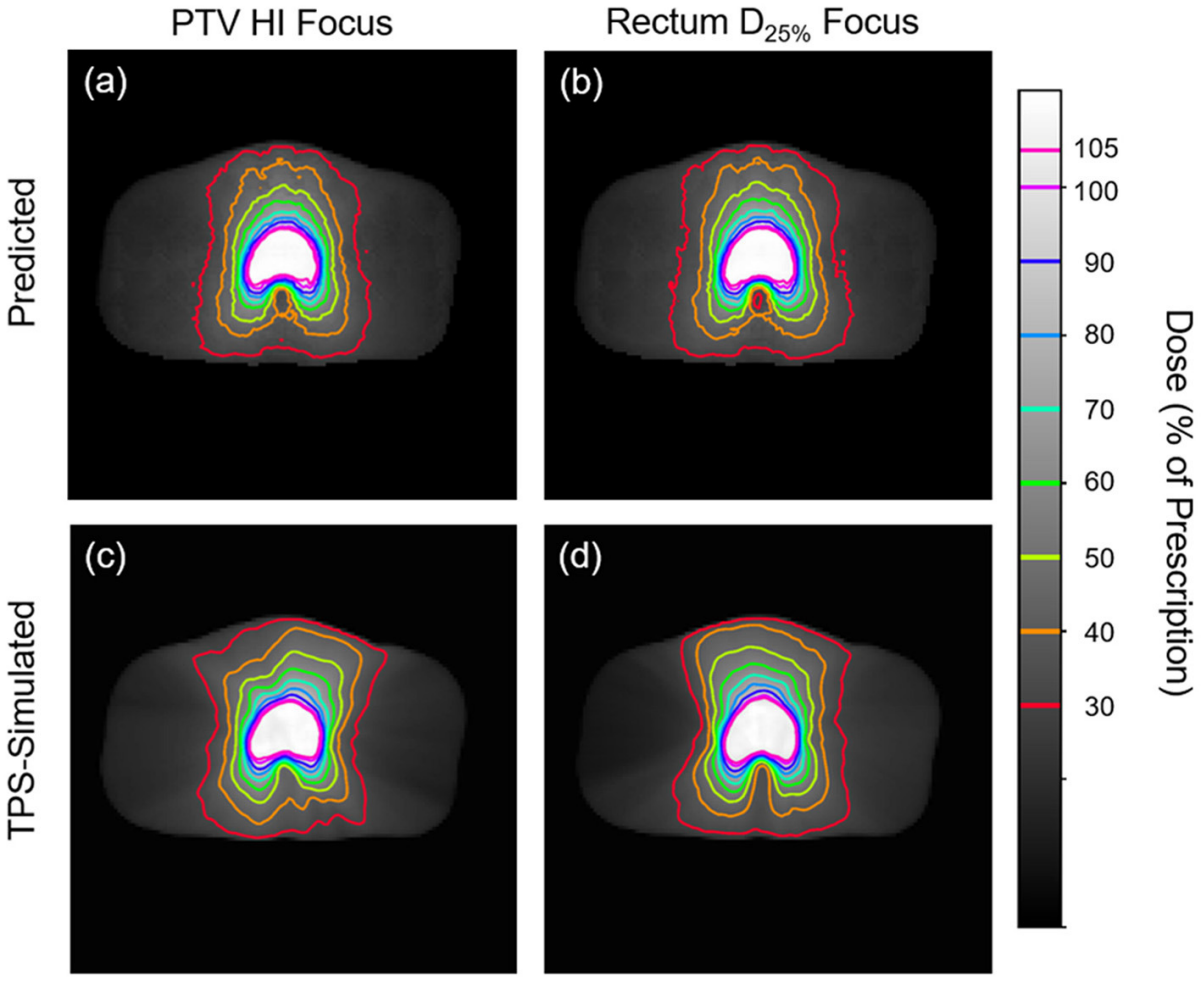
Side-by-side comparisons between the effect of prioritizing PTV HI **(a,c)** or prioritizing rectum D_25%_ **(b,d)** in a dose map prediction **(a,b)** and its corresponding TPS-simulated dose map **(c,d)**. Transverse slices are taken from the center of the PTV, and the patient was randomly sampled from the testing dataset.

**Figure 6:**
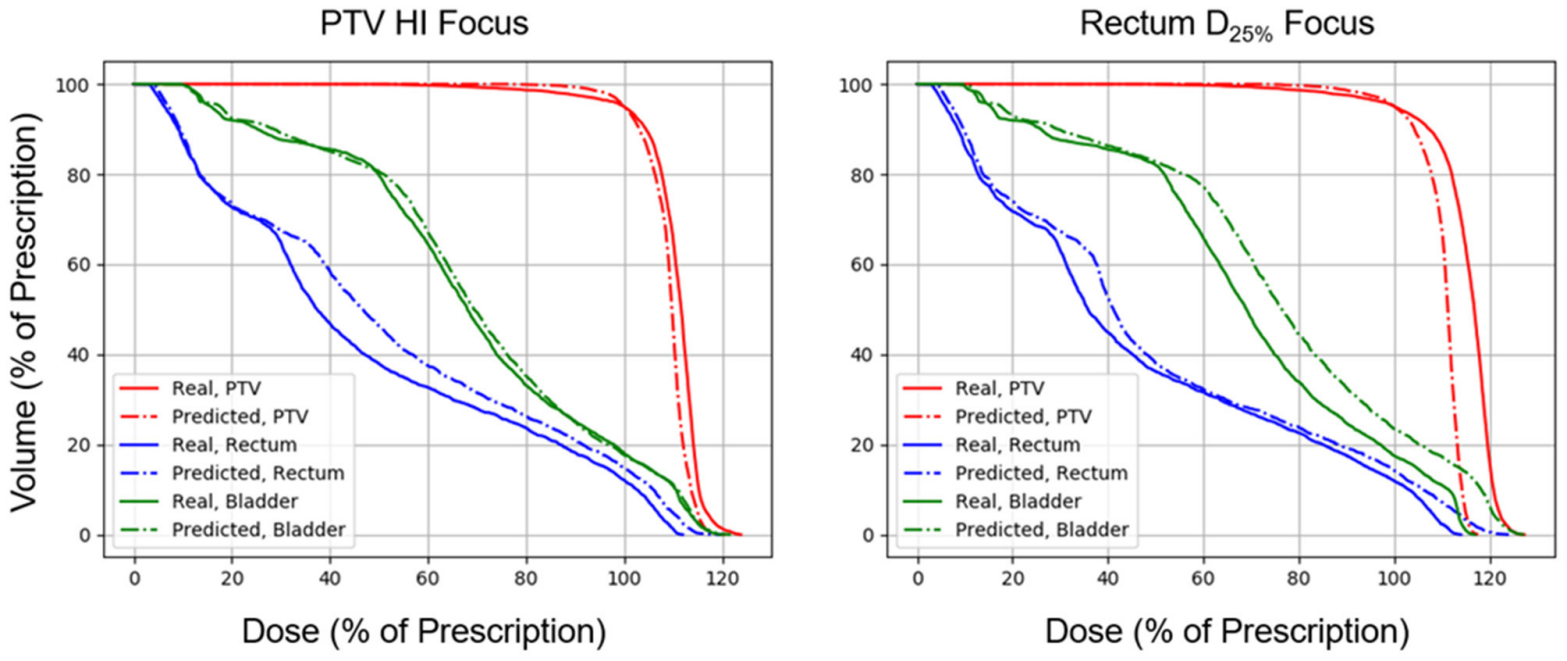
Side-by-side comparisons between the effect of prioritizing PTV HI or prioritizing rectum D_25%_ in a DVH prediction and its corresponding TPS-simulated DVH.

Figures 5, 6 show side-by-side comparisons between the effect of prioritizing PTV HI or prioritize rectum D_25%_ in a dose map prediction and its corresponding TPS simulation. Visually, we can see that the dose map predictions are jagged compared to their respective TPS-simulated dose maps, specifically around the 30% isodose line. We expect this to be the case because the neural architecture of the network does not explicitly promote local smoothness in the dose distribution predictions. However, real dose distributions tend to be smooth and continuous, so that the artificial jaggedness in the prediction of our model is a drawback reflecting the artificial nature of the model. Note that the jaggedness makes the isodose lines look dissimilar, but the general location of the isodose lines corresponds much more strongly to voxel-by-voxel error than the precise shape of the isodose lines. Additionally, we see that the region of the largest isodose displacement is the low dose region anterior to the PTV. Note that this region is not near the PTV or the surrounding critical structures, so that the inaccuracy in this region has a small impact on the predicted PTV/OAR doses.

**Figure 7:**
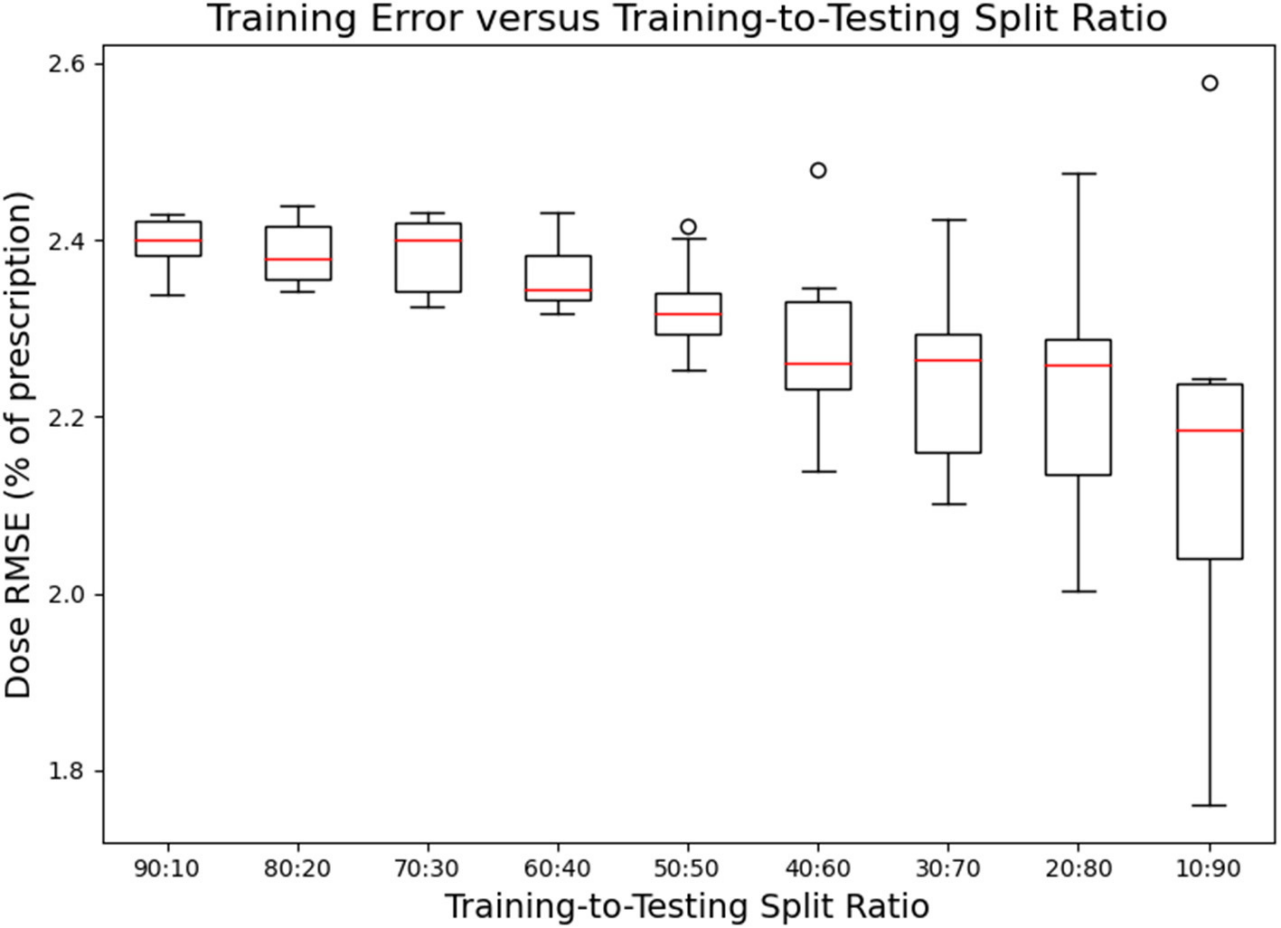
Box-and-whisker plots representing the training set errors of the model as a function of decreasing training-to-testing set split ratios. Each box-and-whisker plot represents the aggregate errors from one split of 10-fold cross-validation.

**Figure 8:**
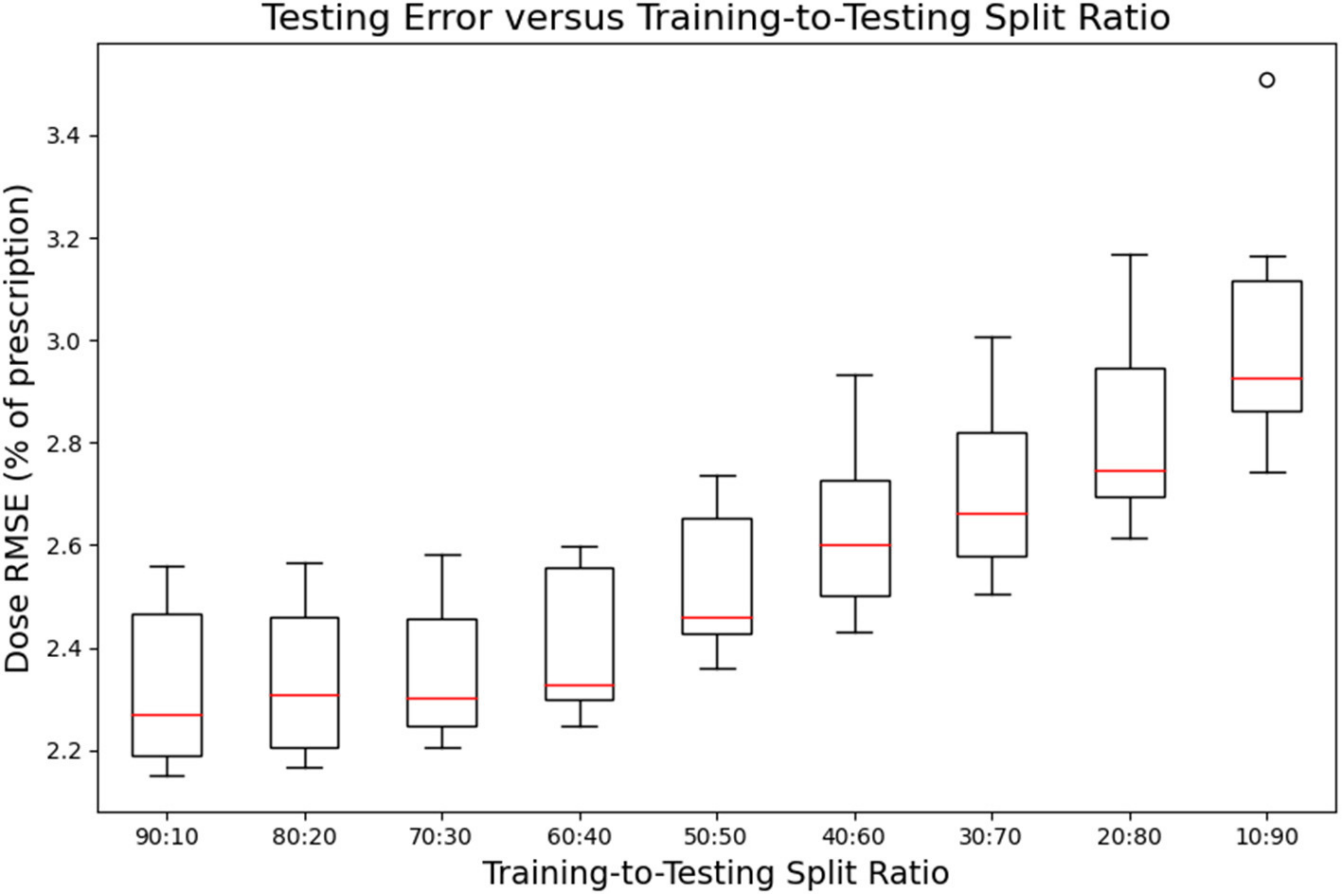
Box-and-whisker plots representing the testing set errors of the model as a function of decreasing training-to-testing set split ratios. Each box-and-whisker plot represents the aggregate errors from one split of 10-fold cross-validation.

Figures 7, 8 show the performance of the model on training and testing sets as a function of the ratios of training set data to testing set data, so that they show the effect of decreasing the number of patients used to train the model. Figure 7 shows that the training set errors decrease as training set size decreases. On the other hand, Figure 8 shows that the testing set errors increase as training set size decreases. From these results, we can see that the performance of the model degrades slightly as the amount of training data shrinks because it increases overfitting to the training data. It is difficult to conclude from these figures exactly how much data is needed to properly fit to the data, but that number is likely between 60 and 90 patients based on the figures (though this number might not generalize to other treatment sites). Figures 7, 8 also suggest that the spread of errors becomes larger with smaller training data sizes, indicating that the performance of the model is more random with smaller training data sizes.

### Model Evaluation Time

Total dose prediction requires an average of 1.26 s to evaluate an entire 25-plan Pareto surface for one patient, or just 0.05 s per plan. This is significantly faster than current commercial dose optimization and dose calculation engines, which can take ~5–30 min per plan. Due to this speed, we anticipate that this model can be used in real-time without needing to interpolate plan doses from a set of previously predicted doses.

### Predicted Pareto Surface Evaluation

The mean Pareto space RMSEs were 10.33 ± 3.57% and 10.11 ± 4.61% for the training and testing sets, respectively, when aggregated over the fifty splits of 10-fold cross-validation. These errors indicate that the training and testing set dose predictions have similar distances to their corresponding TPS-simulated doses in objective space. This contrasts the dose map RMSEs for the training and testing set, which were more dissimilar than the Pareto space RMSEs. Note that the Pareto space RMSE combines the errors across the objectives *via* accumulation rather than averaging, so we expected these numbers to be significantly larger than dose map RMSE.

The mean Pareto space Hausdorff distances were 14.98 ± 5.91% and 14.79 ± 5.77% for the training and testing set dose predictions, respectively, when aggregated over the fifty splits of 10-fold cross-validation. These errors are notably larger and have more variance than the corresponding Pareto RMSEs. However, Hausdorff distances, in general, are more sensitive to outliers than set averaged RMSEs, so we expect this increased magnitude and variance. We see that the training and testing set Hausdorff distances are also similar, indicating that the errors of our model primarily occur at low-dose regions away from the PTV and at critical structures.

The mean Pareto space APDs were 10.17 ± 3.52% and 9.81 ± 4.74% for the training and testing set dose predictions, respectively, when aggregated over the fifty splits of 10-fold cross-validation. These results confirm that the model fitting is not significant, as the training and testing sets had comparable projected distances. As expected, the Pareto APDs are slightly lower than the Pareto RMSEs and Pareto space Hausdorff distance.

The mean Pareto space ANPDs were 8.44 ± 3.29% and 8.85 ± 4.21% for the training and testing set dose predictions, respectively, when aggregated over the fifty splits of 10-fold cross-validation. The ANPD results demonstrate that the performance of the model in Pareto space is similar for both training and testing set predictions. As expected, the Pareto ANPDs are lower than the three other distance metrics because the minimal distance between Pareto surface interpolations tends to be lower than the distance between their vertices.

## Discussion

In this work, we have presented a novel machine learning dose prediction model which takes optimization objective priorities into account, allowing for indirect Pareto surface estimation. Our results indicate that the model can predict doses with good accuracy, as the predicted dose map RMSEs have few percentages of their corresponding TPS-simulated doses. These dose map RMSEs are less than the maximum error tolerance proposed by the ICRU and AAPM TG 142, suggesting that our predictions may be appropriate for clinical dose distribution estimation. Moreover, the model produces just a dose distribution without actually creating a plan, so the model requires a final real plan optimization and dose calculation which will correct these dose map prediction errors prior to treatment delivery. This means that the error in the results of our model only affects treatment planning and not treatment delivery. The evaluated Pareto surface metrics indicate that these dose map predictions make reasonable translations in Pareto space. Our results also indicate that the overfitting of the model to training data dose map RMSE is modest because the training and testing errors are similar.

The prediction speed of our model is particularly encouraging. By predicting each plan in ~0.05 s, our model may be used for real-time treatment planning without needing to interpolate between previously sampled points, allowing the treatment planner to very quickly estimate the doses produced by a given optimization priority combination. This indirectly gives the planner more time to plan per patient, which may improve the quality of the final plan. Moreover, our model only requires patient anatomy and optimization priorities, so it can generate samples from the Pareto surface automatically. This is potentially useful for large-scale automatic theoretical dosimetric investigations of new treatment planning paradigms, such as testing the effects of pushing a dose limit past its historical value or determining the feasibility of treating new structures. More research is needed to investigate these possibilities.

We believe that the speed, accuracy, and proper fitting of our model are due to the design of the model. The implementation of a dose initialization combined with a residual neural network is a novel proposal that appears to model the dose prediction process well. Also, the combination of contiguous and atrous patches during contour processing increases the effective receptive field size of each layer in the ResNet. Achieving a similar effective field-of-view in a more traditional convolutional neural network would involve either increasing the size of each convolution kernel or adding many more layers to the network. However, both of these options involve more model parameters, have increased computational requirements, and are more prone to overfitting. The patch extraction process of our model innovates by incorporating local and global information within each layer without increasing computational requirements or promoting overfitting.

Despite its potential advantages, our model has some limitations which hinder its accuracy and utility. The dose initialization of our model assumes an isotropic inverse exponential decay of dose as a function of inter-slice and intra-slice distances from the PTV. Although this assumption is only appropriate for VMAT plans which involve beam arcs wrapping nearly 360° around the patient, it is likely that other forms of dose initialization exist which are appropriate for IMRT or VMAT with significantly fewer than 360° per arc. Additionally, the model required several hyperparameters (i.e., 6 residual blocks in the neural network, 100 output units for the first two layers in each block, atrous rates of 1, 3, and 10 in patch sampling, etc.), and it is not immediately clear how to determine the optimal values for these hyperparameters aside from trial and error. However, we expect that slight adjustments from our chosen values for the hyperparameters should not significantly change the model performance. Finally, since the output of the model is a dose distribution without an actual plan optimization or dose calculation, the model can only be used to determine the subjectively optimal optimization priorities, which then need to be used in a real plan optimization and dose calculation to actually create a deliverable plan.

This study itself also has several shortcomings that make it difficult to be certain of the performance and generalizability of the model. Due to time constraints and the size of the dataset, it was not feasible to compute the gamma index passing rates of the plans predicted by our model. Gamma index analysis could be useful for confirming the quality of our results, and future research should seek to include this data. However, gamma indices are more generous than their dose difference thresholds (typically 3%, which is higher than our model's performance of 2.42%), so we anticipate that the gamma index passing rates of our data would be quite high. Also, gamma passing rates have been shown to increase in the presence of random noise, so we anticipate that the slight noisiness of our data makes gamma passing rates less useful.

It is also difficult to confirm whether these results would extend to other treatment sites. The dataset in this study is likely large enough to sufficiently represent the population of prostate VMAT treatment cases because of the similarity between our training and testing set errors. This coincides with our expectations because the relevant anatomical structures of prostate cases all tend to be somewhat similar. However, it is not immediately clear how this result is generalizable with other training sets, which may experimentally find that they require more or less training data. Also, this study does not include treatment planning data from other treatment sites, so it is difficult to determine whether this model would generalize well to model another treatment site. Further research needs to be done to test this model on other treatment sites. In particular, the current structure of the model is not built to process the data from multiple treatment sites concurrently. However, it is feasible to modify the structure of this model to learn from multiple treatment sites by incorporating the structure maps alongside their DVH constraints. Further research needs to be done to test these claims.

We have implemented several metrics for evaluating the error between a predicted Pareto surface and its corresponding TPS-simulated Pareto surface. Our metrics reported similar values around 8–15% of dose prescription for both training and testing sets. Again, it is worth noting that these metrics accumulate the errors from each dimension rather than averaging them, which is why these surface metrics are significantly larger than the dose map RMSE of 2–3%. Most of these metrics have an inherent limitation in that they measure errors from the matched pairs of plans which sample their respective surfaces rather than measure errors from the surfaces themselves. Of the metrics presented, we hypothesize that the ANPD is the most appropriate of these metrics due to its use of point-by-point nearest distances between the surfaces, which likely reflects the actual distance between the Pareto surfaces. However, a more theoretical investigation is required to justify the ANPD here as the appropriate metric. Our results show that these metrics are significantly different from each other, which provides evidence that there exists an optimal metric to represent the distance between Pareto surfaces. Also, to our knowledge, no other body of research has applied Pareto space metrics to evaluate the Pareto surfaces of radiation therapy dose predictions. This prevents us from comparing our Pareto space results with previous dose prediction research. To account for this, we have included all these metrics for ease of comparison with future research.

## Conclusion

We have presented a novel machine learning dose prediction model which takes optimization objective priorities into account. The error of the model is modest when applied to our prostate VMAT cases, with average dose map RMSEs of 2.42 ± 0.47% overall patients and all optimization priority combinations in the patient testing set. This model may be used for quickly estimating the Pareto set of feasible dose objectives, which may directly accelerate the treatment planning process and indirectly improve the final plan quality by allowing more time for plan refinement. Future research needs to be done to determine the generalizability of this model to other treatment sites and datasets.

## Data Availability Statement

The raw data supporting the conclusions of this article will be made available by the authors, without undue reservation.

## Author Contributions

PJ was responsible for conducting the bulk of the research and manuscript writing. JZ provided feedback and suggestions for the scientific work to the primary author during research. BK provided clinical feedback regarding the data produced in this research. QW oversaw the research process. All authors contributed to the article and approved the submitted version.

## Conflict of Interest

The authors declare that the research was conducted in the absence of any commercial or financial relationships that could be construed as a potential conflict of interest.

## Abbreviations

VMAT: volumetric modulated arc therapy
MCO: multi-criteria optimization
TPS: treatment planning system
PTV: planning target volume
IMRT: Intensity-modulated radiation therapy
HI: homogeneity index
ResNet: residual network
RMSE: root-mean-square error
APD: average projected distance
ANPD: average nearest point distance
ICRU: International Commission on Radiation Units and Measurements
AAPM: the American Association of Physicists in Medicine
FC: fully connected
L-ReLU: leaky rectified linear unit
SS: scaled softsign.
